# shinyOPTIK, a User-Friendly R Shiny Application for Visualizing Cancer Risk Factors and Mortality Across the University of Kansas Cancer Center Catchment Area

**DOI:** 10.1200/CCI.21.00118

**Published:** 2022-01-11

**Authors:** Qing Xia, Dinesh Pal Mudaranthakam, Lynn Chollet-Hinton, Ronald Chen, Hope Krebill, Hanluen Kuo, Devin C. Koestler

**Affiliations:** ^1^ Department of Biostatistics & Data Science, University of Kansas Medical Center, Kansas City, KS; ^2^ The University of Kansas Cancer Center, Kansas City, KS; ^3^ Department of Radiation Oncology, University of Kansas Medical Center, Kansas City, KS

## Abstract

The University of Kansas Cancer Center (KU Cancer Center) recently developed a data warehouse to Organize and Prioritize Trends to Inform KU Cancer Center (OPTIK). The OPTIK database aggregates and standardizes data collected across the bistate catchment area served by the KU Cancer Center. To improve the usability of the OPTIK database, we developed *shinyOPTIK*, a user-friendly, interactive web application for visualizing cancer risk factor and mortality rate data across the KU Cancer Center Catchment area.

**METHODS:**

Data in the OPTIK database were first consolidated at the county level across the KU Cancer Center catchment area. Next, the *shinyOPTIK* development team met with the KU Cancer Center leadership to discuss the needs and priorities of the *shinyOPTIK* web application. *shinyOPTIK* was developed under the R Shiny framework and consists of a user interface (ui.R) and a web server (server.R). At present, s*hinyOPTIK* can be used to generate county-level geographical heatmaps; bar plots of demographic, screening, and risk factors; and line plots to visualize temporal trends at different Rural-Urban Continuum Codes (RUCCs), rural-urban status, metropolitan, or county levels across the KU Cancer Center catchment area.

**RESULTS:**

Two examples, adult obesity prevalence and lung cancer mortality, are presented to illustrate how researchers can use *shinyOPTIK*. Each example is accompanied by post hoc visualizations to help explain key observations in terms of rural-urban disparities.

**CONCLUSION:**

Although *shinyOPTIK* was developed to improve understanding of spatial and temporal trends across the population served by the KU Cancer Center, our hope is that the description of the steps involved in the creation of this tool along with open-source code for our application provided herein will serve as a guide for other research centers in the development of similar tools.

## BACKGROUND

The University of Kansas Cancer Center (KU Cancer Center) serves a population that extends across rural and urban counties of Kansas and 18 counties of western Missouri that lay along the state border. Monitoring population-level demographic, screening, risk factor, and cancer incidence and/or mortality data across the bistate catchment area requires data merging and normalization across state-level data resources. In addition, the changing population pattern across the state of Kansas complicates the assessment of cancer needs throughout the catchment area. For example, population growth in Kansas has not been uniform across the state.^1^ Although the population of urban counties (counties with a population density of ≥ 150 persons/square mile) in the state of Kansas has increased by 80.8% from 1960 to 2016, the population of rural (6-19.9 persons/square mile) and frontier counties (< 6 persons/square mile) decreased by 22.2% and 41.1%, respectively, across the same time period. Moreover, all the population growth in the state of Kansas between 2000 and 2016 was among minority populations.^1^ Furthermore, using County Health Rankings data by Robert Wood Johnson Foundation (2021), substantial health disparities have been observed across the KU Cancer Center catchment area, particularly two adjacent and highly populous counties in Kansas. Johnson County, Kansas, ranks number one in health outcomes, health behaviors, and clinical care, whereas the adjacent Wyandotte County ranks last (104th) in health outcomes, last in health behaviors, and 91^st^ in clinical care across the state.^2^

**CONTEXT**

**Key Objective**
Development of a user-friendly, interactive web application to facilitate a better understanding of cancer risk factors and mortality trends across a Cancer Center catchment area.
**Knowledge Generated**
We describe the development of *shinyOPTIK*, an interactive web application that can be used generate county-level geographical heatmaps; bar plots of demographic, screening, and risk factors; and line plots to visualize temporal trends across the University of Kansas Cancer Center (KU Cancer Center) catchment area. We illustrate the use of this tool to describe temporal and spatial trends in the prevalence of adult obesity prevalence lung cancer mortality across the population served by the KU Cancer Center.
**Relevance**
Applications like *shinyOPTIK* have the potential to assist cancer centers in monitoring trends in their catchment area and can be extremely useful tools in resource allocation and defining priorities.

Ongoing assessment of trends related to screening and risk factors, cancer incidence and mortality, and access to care is needed to strategically focus research and outreach efforts. To meet this need, the KU Cancer Center recently developed a data warehouse to Organize and Prioritize Trends to Inform KU Cancer Center (OPTIK)^3^ and an accompanying Tableau server^4^ to generate heat maps, bar graphs, pie charts, and other visualizations of cancer risk factors and mortality rates across the catchment area. The OPTIK database was initially linked with a Tableau Server for data exploration and visualization. Tableau is an interactive and intuitive data visualization platform that is amenable for a variety of data types, both structured and unstructured. Despite the many positive aspects of the Tableau platform, it has several limitations in terms of its application to the OPTIK database. First, Tableau requires a license for users to acquire data visualizations. Second, Tableau is not a training-free application, which presents a logistical obstacle in the widespread utilization of the OPTIK database. Finally, Tableau has limited statistical modeling capabilities, which can be an impediment to implementing formal statistical analyses on catchment area data.

In this article, we introduce a user-friendly, interactive web application, *shinyOPTIK*, to improve the usability of the OPTIK database and in doing so, facilitate a better understanding of cancer risk factors and mortality trends across the KU Cancer Center catchment area. The *shinyOPTIK* web application was developed using the R Shiny platform, which we believe offers three distinct advantages over the existing Tableau format: (1) the user interfaces that one can develop through R Shiny are easy to use and do not require any a priori training, (2) R Shiny is freely accessible and does not require a license, and (3) because R Shiny is based in the R statistical programming language, statistical modeling capabilities can be seamlessly integrated within the web application, including novel and recently published statistical methods with corresponding R packages that contain functions to implement such methods. In its current form, *shinyOPTIK* is aimed at data exploration and hypothesis generation and uses the OPTIK database to generate county-level geographical heatmaps, bar plots of demographic, screening, and risk factors from 2005 to 2017, and all cancer type–specific mortality data at two 5-year periods from 2008 to 2017. Temporal trends for all data are visualized by a series of line plots by Rural-Urban Continuum Code (RUCC) region, rural-urban status, metropolitan, or county. In what follows, we describe the process of developing *shinyOPTIK*, its architecture, and key functionalities and present two examples where *shinyOPTIK* was used to discern interesting features of the KU Cancer Center catchment area. We finish with a discussion of the highlights, limitations, and future initiatives for *shinyOPTIK* and offer advice to researchers who are interested in the creation of similar visualization and data exploration tools.

## METHODS

### Process of Developing *shinyOPTIK*

The development and refinement of *shinyOPTIK* was an iterative process that involved a series of steps. First, data from the bistate KU Cancer Center catchment area (see the Data Supplement and Sources of Data for the OPTIK Database) were consolidated at the county level.^3^ After consolidating data, the *shinyOPTIK* development team met with KU Cancer Center leadership, including members of the Community Outreach and Engagement group, to discuss the needs and priorities of the *shinyOPTIK* web application. For example, the development team solicited suggestions on what specific data and variables to include in the application, how to best organize and visualize those data across the catchment area, specific features to improve and enhance its usability and user-friendliness, etc. These meetings took place at regular intervals throughout the development of *shinyOPTIK* to help ensure that the application was easy to use and met the needs of its target userbase in terms of what data were being visualized and how they were being visualized. The web-based visualization application was implemented under the R Shiny framework within the RStudio integrating development environment for the R statistical programming language.^5^ The R Shiny interface for *shinyOPTIK* consists of two parts: (1) a user interface (ui.R) that allows users to specify the input parameters (eg, what variable(s) to visualize, time frame for visualization, region level, etc) and (2) a web server (server.R) that executes the R scripts on the basis of the input from ui.R and returns the output to the web interface. Both ui.R and server.R were written by the *shinyOPTIK* development team and depend on the following R packages: shiny, ggplot2, tidyverse, gridExtra, usmap, urbnmapr, shinydashboard, dplyr, data.table, grid, and broom. In addition, ggplot2 and usmap were used to generate bar or line plots and geographic heatmaps, respectively. Although the present version of *shinyOPTIK* available to the public is intended to be used for data exploration and hypothesis generating and does not presently include advanced statistical modeling capabilities, statistical methods for conducting formal statistical tests on the basis of data in the OPTIK database can be seamlessly integrated within the existing tool due as shown in the Data Supplement and Statistical Modeling.

### Architecture of *shinyOPTIK*

**FIG 1.**
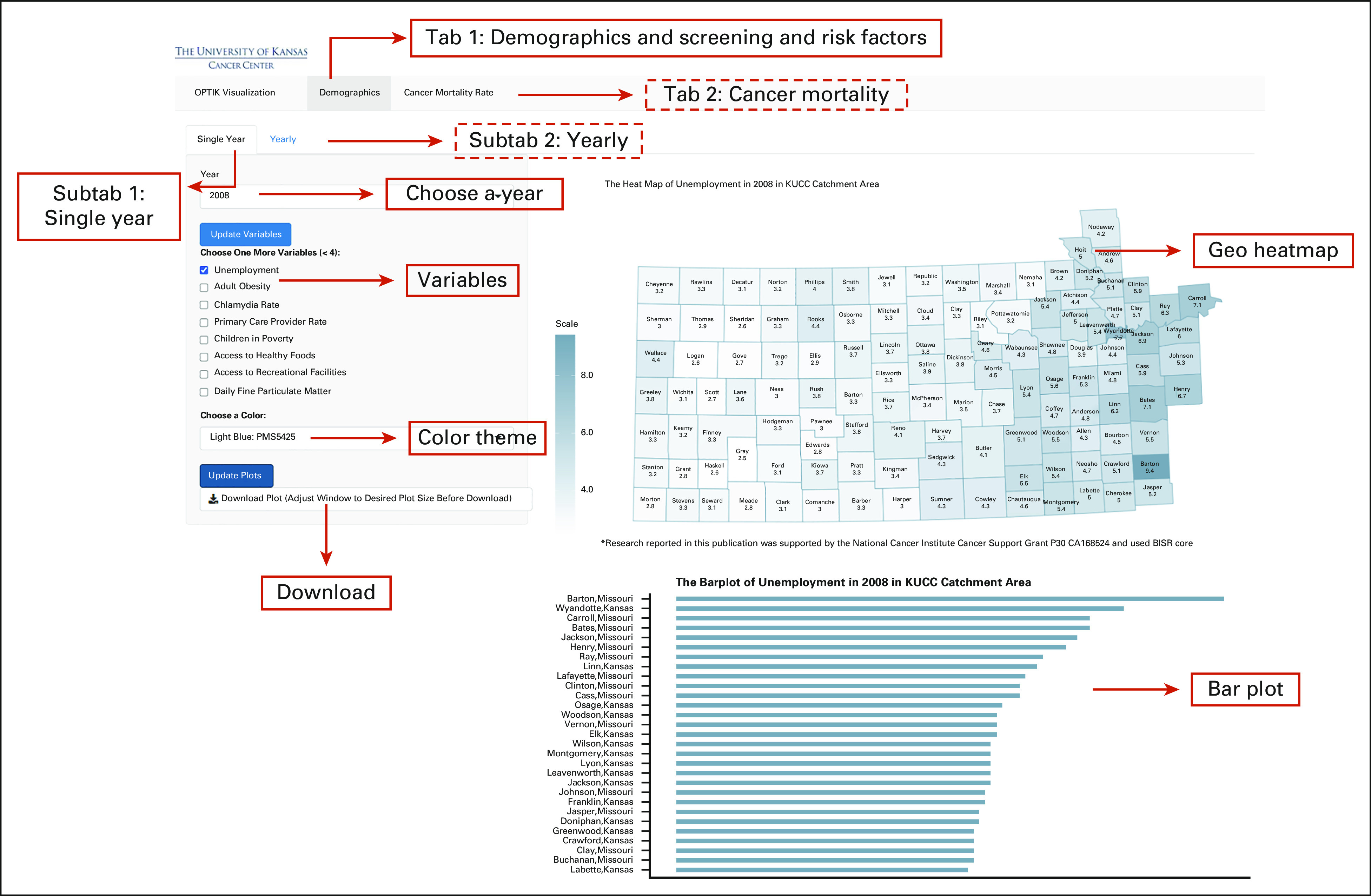
Interface of *shinyOPTIK*—Demographics tab. Solid boxes show the contents in the screenshot, and dashed boxes indicate the features of *shinyOPTIK* that are not shown in this figure. KUCC, The University of Kansas Cancer Center; OPTIK, Organize and Prioritize Trends to Inform KU Cancer Center.

**FIG 2.**
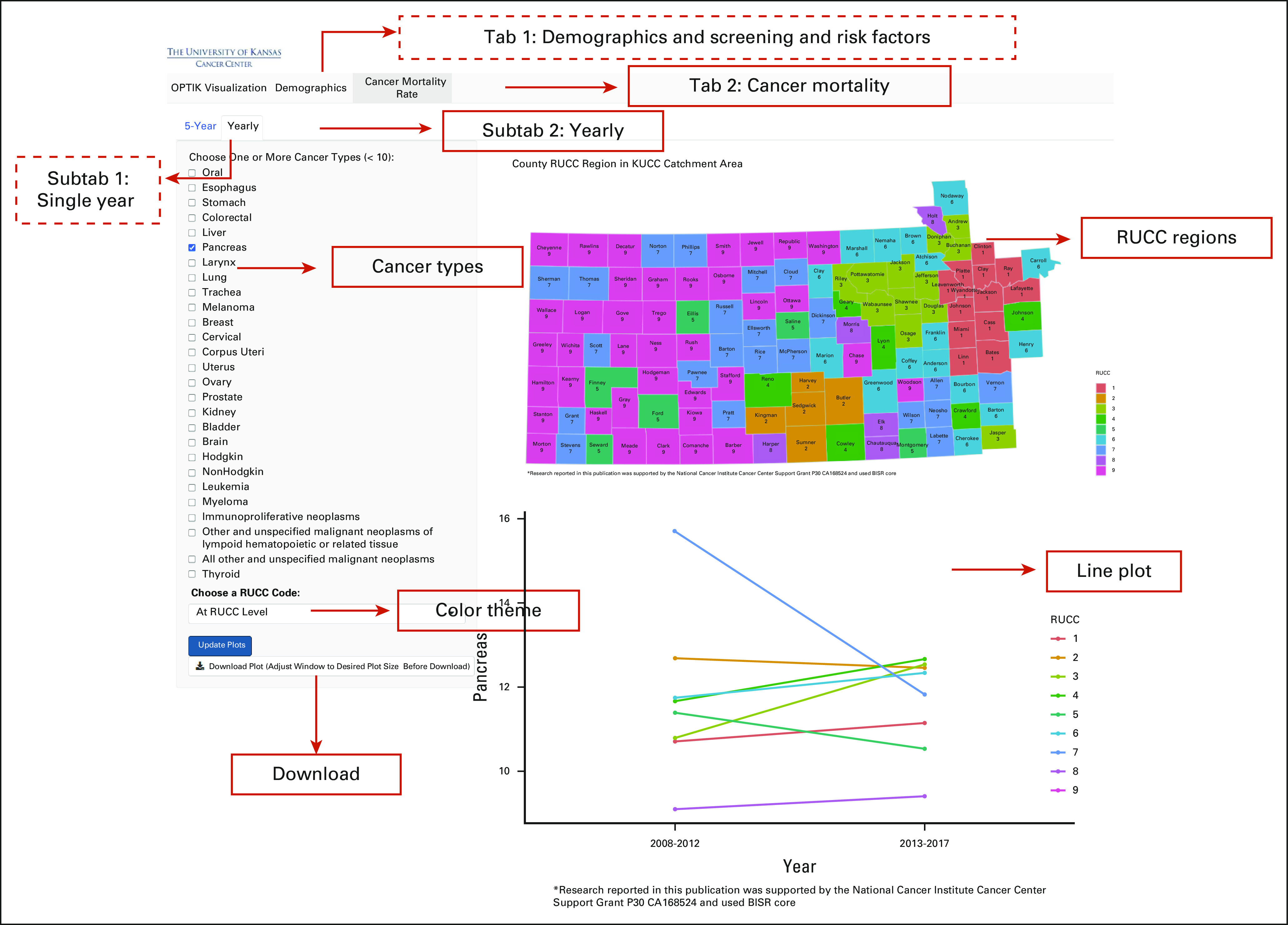
Interface of *shinyOPTIK*—Cancer Mortality tab. Solid boxes show the contents in the screenshot, and dashed boxes indicate the features of *shinyOPTIK* that are not shown in this figure. KUCC, The University of Kansas Cancer Center; RUCC, Rural-Urban Continuum Code.

At present, *shinyOPTIK* consists of two tabs: Demographics and Cancer Mortality Rates. Figures 1 and 2 depict the contents and features of the Demographics and Cancer Mortality Rate tabs, respectively. In the following sections, we discuss the scope of visualizations that are possible for Demographics and Cancer Mortality Rate tabs. Table 1 shows a list of all available variables and cancer types (for the Cancer Mortality Rate tab) that users can select in *shinyOPTIK*. All graphs generated by *shinyOPTIK* can be downloaded as PDFs for future use in manuscripts and/or grant applications.

**TABLE 1.**
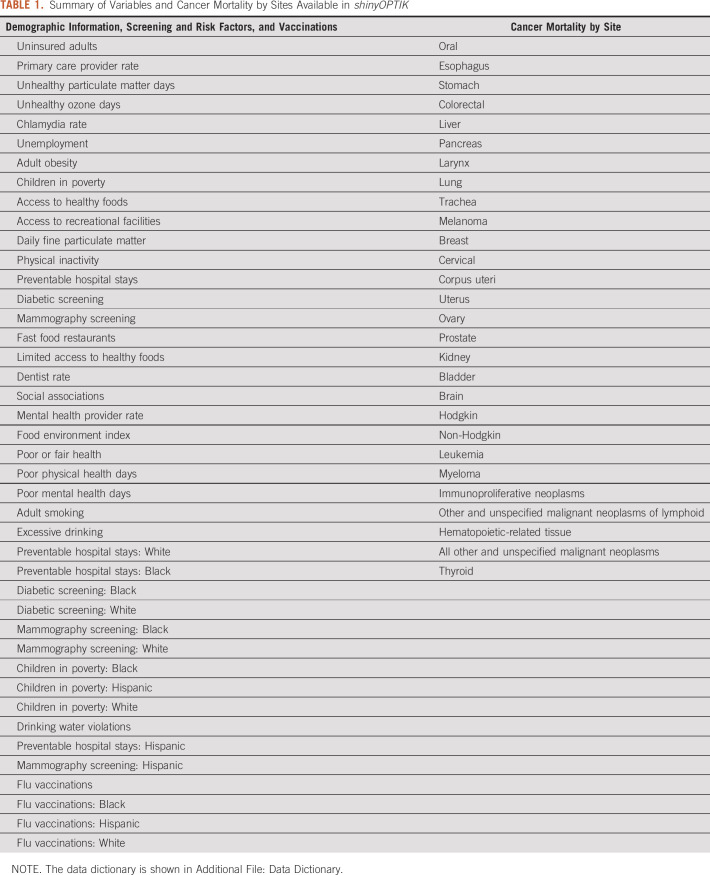
Summary of Variables and Cancer Mortality by Sites Available in *shinyOPTIK*

Demographics is the default tab when launching *shinyOPTIK*. As depicted in Figure 1, data visualizations are fulfilled in two subtabs: (1) Single Year and (2) Yearly. In Single Year, data related to demographic information, screening and risk factors, vaccinations, etc, are visualized at the county level for a single year from 2005 to 2017. After choosing a specific year and outcome variable(s) of interest, *shinyOPTIK* generates geographical heatmaps to display county-specific values on a map of the KU Cancer Center catchment area. Counties with low and high values are colored based on a monochromatic color scale, allowing the user to understand spatial variation and distribution of outcomes across the catchment area and helping users identify counties that exhibit unusually high or low values. Text labels for each county along with the value of the selected variable(s) are included on geographical heatmaps. In addition to geographical heatmaps, a bar plot is generated and displayed below the heatmap, providing users with an alternative way of visualizing the data and facilitating easier discernment of counties with high or low values of the selected variable(s). For the Yearly subtab, data are aggregated by taking the mean of counties within a region, eg, rural-urban level, metropolitan level, and Rural-Urban Continuum Code (RUCC) classification scheme.^6^ Line plots are created on the basis of the aggregated mean data for each year at different region levels (see below for more details) and can be used for understanding temporal changes for the selected outcome variable(s) of interest.

Cancer Mortality Rate is the second tab in *shinyOPTIK* (Fig 2). Mortality rates are age-adjusted and given as the number of deaths attributed to specific cancer type per 100,000 population. Similar to the Demographics subtab, data visualizations of cancer mortality are also available via two subtabs: (1) 5-Year and (2) Yearly. In the 5-Year subtab, the mortality rates of a total of 27 different cancer types at two 5-year periods from 2008 to 2017 are available at the county level across the KU Cancer Center catchment area. Geographical heatmaps describe the spatial distribution of cancer mortality rates at the county level to assist users in identifying counties with high or low age-adjusted mortality rates. Darker color shades indicate higher mortality rates, and lighter shades, lower mortality rates. Text labels, including county and estimated mortality rates, are included on the geographical heatmaps. In addition, bar plots are used to sort counties by their mortality rate, from those with high values to those with low values. To protect patient privacy and be compliant with Kansas Department of Health and Environment policy, counties with mortality rates on the basis of < 16 reported cases are suppressed at the county level and not visualized. This suppression rule comes from the NCHS and CDC; see Suppression for Confidentiality at Division of Cancer Prevention and Control, Centers for Disease Control and Prevention.^7^ Temporal changes in mortality rates of different types of cancers are shown in the second subtab, Yearly, at various regional levels, eg, rural-urban level, metropolitan level, and RUCC classifications.

### Region Levels of *shinyOPTIK*

*shinyOPTIK* shows temporal changes by aggregating county-level data into geographical regions on the basis of RUCC, shown in the Data Supplement. RUCCs range from 1 to 9, with the most metropolitan counties categorized as 1 and the most rural counties as 9, while adjusting for adjacency to a metropolitan area (Data Supplement). Rural-urban levels are shown in the Data Supplement, where urban areas are the counties that have a RUCC classification of 1-3 and rural areas are the counties categorized as 4-9 in RUCC. We further aggregated counties of catchment areas into urban (RUCC = 1, 2, 3), adjacent rural (RUCC = 4, 5, 6), and nonadjacent rural (RUCC = 7, 8, 9; Data Supplement).

## RESULTS

To illustrate how researchers can use the *shinyOPTIK*, we present two examples: adult obesity prevalence and lung cancer mortality. Each example is accompanied by post hoc visualizations to help explain key observations in terms of rural-urban disparities, which refers to a significant disparity in the overall rate of disease incidence, prevalence, morbidity, mortality, or survival rates.^8^ Many studies have reported that residents in rural communities have less access to cancer prevention strategies and/or interventions, regular screening, and other catalyzing efforts that contribute to increased life expectancy and healthier populations as compared with urban residents, resulting in rural-urban disparities in health behavior and status.^9-11^

### Example 1: Adult Obesity Prevalence Increases Over Time and Is Markedly Higher in Rural Versus Urban Areas

**FIG 3.**
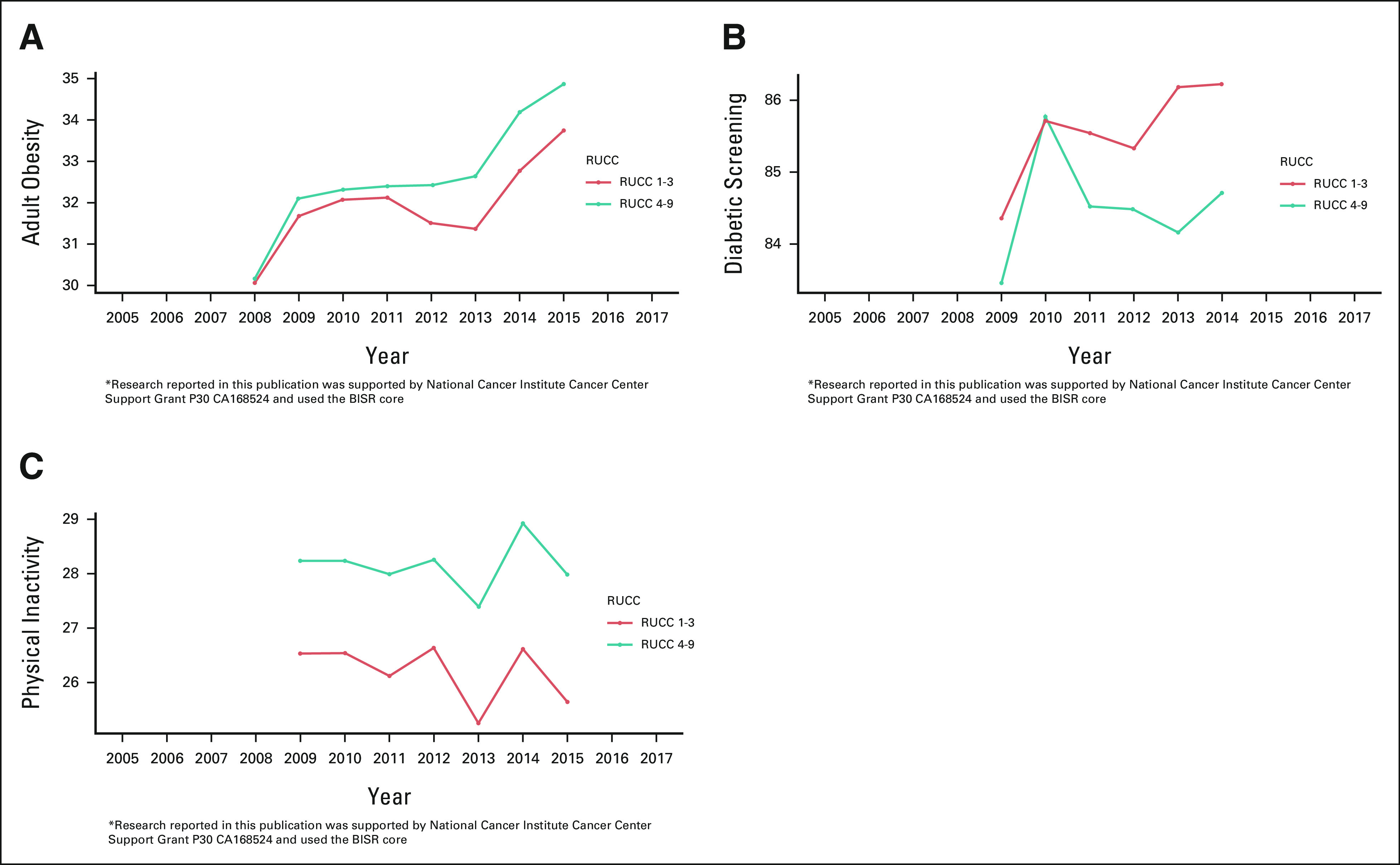
Line plots of yearly estimates of (A) adult obesity and behavioral determinants (eg, (B) diabetic screening and (C) physical inactivity) at the urban/rural level in the KUCC catchment area across 2008-2015. KUCC, The University of Kansas Cancer Center; RUCC, Rural-Urban Continuum Code.

Rural residents tend to have higher rates of chronic diseases compared with urban residents, and obesity may be a major contributor to the urban-rural disparity.^12^ Figure 3A shows an increase in adult obesity prevalence in both urban and rural areas across the KU Cancer Center catchment area from 2008 to 2015. We observe a higher prevalence of obesity in rural compared with urban areas, and the rural-urban disparity in obesity prevalence appears to increase over time. To understand what might be driving our observations in obesity prevalence, we examined the temporal pattern of behavioral determinants (eg, physical inactivity, calculated as percentage of adults age ≥ 20 years and reporting no leisure-time physical activity), as areas with reduced physical activity and those with a higher daily energy intake or percent kcal from fat have been extensively shown to associate with higher obesity prevalence.^13^ In the KU Cancer Center catchment area, adults residing in rural areas were less likely to meet or exceed physical activity recommendations (defined in the study by Piercy et al^14^) as compared with adults living in urban areas, from 2009 to 2015 (Fig 3C). In 2008, access to healthy food of rural residents was 43.4, which was lower than urban residents at 49.0. Furthermore, as obesity is a major risk factor and key prevention target for type 2 diabetes mellitus,^15,16^ we also examined the temporal pattern of the prevalence of diabetic screening between rural and urban areas and observed that urban residents had higher rates of diabetic screening as compared with rural residents from 2009 to 2014 (Fig 3B). Noting the exploratory nature of our analysis, these findings may indicate that obesity and diabetes prevention and management strategies are more prevalent and successful in urban compared with rural counties. Nevertheless, this information motivated the selection of obesity as a priority cancer prevention focus for KU Cancer Center research and community outreach programs.

### Example 2: Changes in Lung Cancer Mortality in the KU Cancer Center Catchment Area

**FIG 4.**
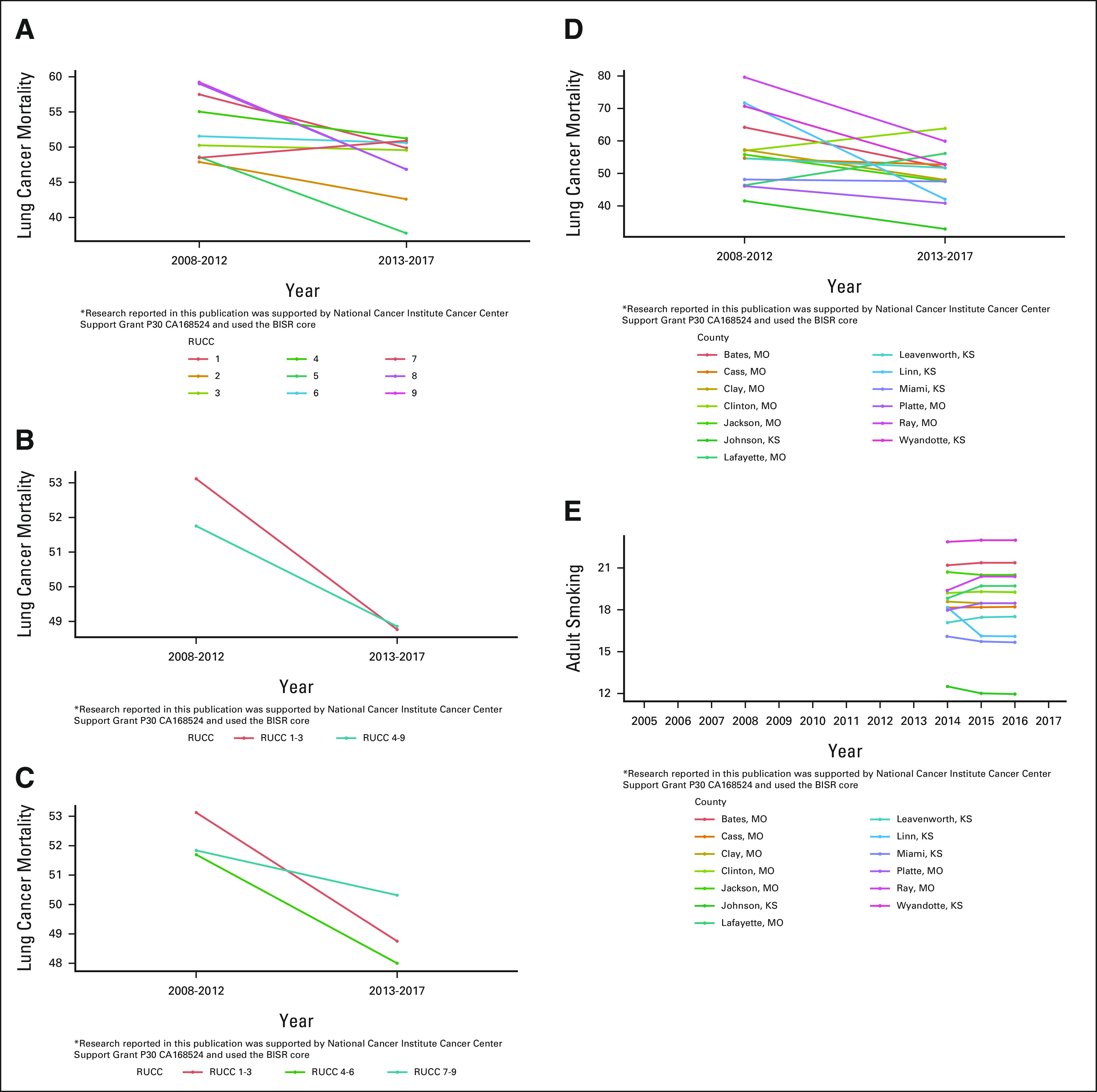
Line plots of age-adjusted mortality rates of lung cancer across two time periods shown at different regional levels: (A) RUCC level, (B) rural/urban level, and (C) metropolitan level and disparity of (D) lung cancer mortality and (E) adult smoking within two adjacent counties with RUCC = 1 in the KUCC catchment area. KUCC, The University of Kansas Cancer Center; RUCC, Rural-Urban Continuum Code.

Lung cancer mortality rates are given at the county level and are defined as the number of deaths caused by lung cancer per 100,000 individuals during the 5-year period (2008-2012 or 2012-2017). We observed a decrease in lung cancer mortality from the 2008-2012 time period to the 2013-2017 period across various regional levels (Fig 4A) and also higher lung cancer mortality rates in aggregated urban compared with rural areas in the 2008-2012 time period (Fig 4B). The observed higher lung cancer mortality in urban areas has also been reported in China^17^ and in the United Kingdom^18^ and is opposite of what has been reported at the national level in the United States, where the lung cancer mortality rates appear to be higher in rural as compared with urban areas.^19,20^ We also observed decreased rural-urban disparities in lung cancer mortality from 2008-2012 to 2013-2017 in Figure 4B, a time frame that coincides with the National Cancer Institute (NCI) designation of the KU Cancer Center in 2012.

Figure 4D shows disparities in lung cancer mortality rates in the 2008-2012 time period between two adjacent counties in Kansas: Johnson County and Wyandotte County. Among counties in Kansas, Johnson County had the lowest lung cancer mortality rates in 2008-2012 and 2013-2017 (41.3; 95% CI, 38.7 to 43.9 and 32.8; 95% CI, 30.8 to 35.0, respectively), whereas Wyandotte County was observed to have one of the highest mortality rates (70.8; 95% CI, 64.7 to 77.3 during 2008-2012 and 52.8; 95% CI, 47.7 to 58.3 during 2013-2017). This disparity may in part be driven by differences in adult smoking rates between counties. On the basis of smoking rates from 2014 to 2016 (Fig 4E), Wyandotte County has the highest smoking rate compared with Johnson County, which has the lowest smoking rate.

## DISCUSSION

The KU Cancer Center recently developed a data warehouse to OPTIK by consolidating data from various private and public data sources to monitor trends in cancer risk factors, surveillance, and mortality rates across its catchment area. This effort is but one example among a growing number where centers and/or institutes have created consolidated databases and corresponding web applications for visualizing cancer risk factor, incidence, and mortality data statewide or across a predefined catchment area.^21-23^ Despite the many strengths of the Tableau platform, the existing platform used to visualize data in OPTIK, it has several limitations for our purposes. To circumvent these limitations, *shinyOPTIK* was developed using the R Shiny platform as a freely accessible web application, with the goal of increasing the widespread use of OPTIK database by members and affiliates of the KU Cancer Center. Piloting *shinyOPTIK* with members of the KU Cancer Center reveals that *shinyOPTIK* is user-friendly, does not require any a priori training, and can be used by individuals from a variety of different backgrounds. We do, however, note that these qualities are not uniformly shared across all R Shiny interfaces as the user-friendliness and the degree to which such applications require a priori training rest on developers of the specific web application, along with the complexity of the data, analyses, and/or visualizations that the web interface was created to address. Moreover, because *shinyOPTIK* is based in the R environment, statistical modeling capabilities can be seamlessly integrated over time and by demand, including novel and recently published statistical methods with corresponding R packages that contain functions to implement such methods. We have carried out a proof-of-principle example, whereby functions for implementing several different statistical tests of demographic variables and cancer mortality rate data were added to *shinyOPTIK* (see the Data Supplement And Statistical Modeling) to illustrate how such tools can be added in future versions of this application. Since *shinyOPTIK* is written completely in R, those familiar with the R programming can modify the R code to include statistical modeling capabilities and analysis methods that meet their needs.

The first step in the development of applications like *shinyOPTIK* is defining the target area or population. In the case of *shinyOPTIK*, the target area or population consists of the bistate catchment area defined by the KU Cancer Center, which includes all counties in the state of Kansas and 18 counties in western Missouri that boarder the state of Kansas. For other NCI-designated cancer centers, the target area or population would be the catchment area defined by a particular cancer center. After determining the target area or population, researchers would next determine what data are needed to meet their purpose and goals (eg, annual cancer-specific incidence and/or mortality rates, cancer screening rates, demographics, etc) and assemble the necessary data sources that contain those variables or end points over the defined target area. For *shinyOPTIK*, data sources included the following: the Kansas and Missouri Cancer Registries, Behavioral Risk Factor Surveillance System, County Health Rankings, Centers for Disease Control and Prevention, US Census Report, and National Immunization surveys, among others (Data Supplement). After the identification of the data source(s) that contain variables or end points of interest, the next step involves the aggregation and consolidation of such database(s).^3^ Because the data sources comprising the OPTIK database include data collected at different geographic levels (eg, census tracts, county, etc), data sets were merged at the county level to offer a level of standardization because of interest in visualizing cancer risk factors and mortality rates at the county level. Once the above steps are completed, the basic framework of *shinyOPTIK* could be adapted, replacing the KU Cancer Center catchment area with the target area or population of the institute or center and modifying the ui.R script that specifies the input parameters (eg, what variable(s) to visualize, time frame for visualization, region level, etc) on the basis of data sources, variables, time frame, etc, of interest to the particular center or institute.

Despite the utility of *shinyOPTIK* and its potential in improving the KU Cancer Center's understanding of its catchment area, it is not without certain limitations. As previously mentioned, the demographic, cancer risk factor, and cancer mortality data used in *shinyOPTIK* are aggregated at the county level and thus lack the granularity that would be required for statistical inference at the individual level. Furthermore, although not a limitation of the web application itself, it deserves mentioning that because cancer incidence data are restricted in Kansas, visualizations that are possible in *shinyOPTIK* are limited to demographic, cancer risk factor, and cancer mortality data at present. Finally, although the OPTIK database is updated annually to include the most recent data, new data sets within *shinyOPTIK* will need to be uploaded manually to coincide with updates to the OPTIK.

Future directions for *shinyOPTIK* primarily revolve around the addition of statistical modeling capabilities to meet the needs of its users. This includes the addition of mixed effects models and/or generalized additive models for characterizing trends in demographic, cancer risk factor, and mortality rates over time, along with the addition of geographically weighted regression models to formally test relationships between demographic, cancer risk factor variables, and mortality in a framework where spatial heterogeneity appropriately accounted for. We further aim to include statistical modeling capabilities for comparing rates of surveillance and other cancer risk factors (eg, smoking) before and after policy changes (eg, Tobacco 21) to understand the impact of policy changes on our catchment area.

In conclusion, we have discussed the development of a user-friendly visualization web application, *shinyOPTIK*, whose goal is to increase the utilization of OPTIK database and by doing so, enhance understanding of the changing trends in cancer risk factors and mortality across the KU Cancer Center catchment area. We have shown how this application can be used to generate meaningful visualizations that can be used as the basis to support further study into temporal and spatial trends in cancer risk factors and cancer-specific mortality rates. Applications like *shinyOPTIK* have the potential to assist cancer centers in monitoring trends in their catchment area and can be extremely useful tools in resource allocation and defining priorities.

## Data Availability

*shinyOPTIK* can be freely accessed at https://optik.shinyapps.io/OPTIK. The code and example data for *shinyOPTIK* can be found at https://www.dropbox.com/sh/htgixo98dttlrzf/AACsQuCDU6aluHsnfTWSLciXa?dl=0 *shinyOPTIK_stats* can be found at https://www.dropbox.com/sh/360qkm9kgnhwpor/AABoBo7XwQ7bYVIBxg3zm1DFa?dl=0.
